# Editorial: Role of Y Chromosome in Molecular Anthropology, Forensics, and Genetic Genealogy

**DOI:** 10.3389/fgene.2022.863455

**Published:** 2022-06-09

**Authors:** Sibte Hadi, Jun Yao, Atif Adnan

**Affiliations:** ^1^ Department of Forensic Sciences, College of Criminal Justice, Naif Arab University for Security Sciences, Riyadh, Kingdom of Saudi Arabia; ^2^ Department of Forensic Genetics, School of Forensic Medicine, China Medical University, Shenyang, China

**Keywords:** Y chromosomal STRs, archaeogenetic, rapidly mutating Y STRs, snps, forensic genetics

The Human Y chromosome has proven to be a potent tool for studying human genetic anthropology due to its haploid state and the presence of a wide variety of markers (Quintana-Murci and Fellous, 2001). The accumulation of mutations has led to the separation of the Y lineages, which have been extensively studied (Quintana-Murci and Fellous, 2001; Knight et al., 2003; Tambets et al., 2004; Yang et al., 2014; Jobling and Tyler-Smith, 2017; Kivisild, 2017). Various markers have been utilised to study populations and lineages over different time scales. This makes the Y chromosome more powerful than mtDNA, which does not harbour the array of markers that the Y chromosome contains (Jobling and Tyler-Smith, 1995). Y chromosome microsatellites (Y-STRs) and single nucleotide polymorphisms (SNPs) have been extensively used in forensic applications and population studies. However, Alu insertion YAP and duplications/deletions have also been useful in population studies (Hammar et al., 1981; Hammer, 1994).

STRs have been extensively employed in forensic analysis and kinship testing and various commercial multiplexes are available. Specifically, Y STRs are used in the analysis of samples in sexual offense casework and various mixture analyses. Two mega multiplexes containing several Y-STRs are Powerplex Y 23 by Promega (Thompson et al., 2013) and Y Filer Plus by Thermo Fisher Scientific (Gopinath et al., 2016). Both kits are employed by forensic laboratories for casework and are used for kinship testing. Among the Y-STRs, a unique variety is Rapidly Mutating (RM) Y STRs, which have a niche application in separating paternally related individuals (Ballantyne et al., 2010; Ballantyne et al., 2012; Adnan et al., 2016; Neuhuber et al., 2022). Similarly, slowly mutating Y STRs (Baeta et al., 2018) might prove complementary to SNP markers in evolutionary studies, though the number of such markers needs to be increased for precise inferences.

Y SNPs like sY81 and SRY were studied initially, followed by the detection of a vast array of such markers (Underhill et al., 2000; Hinds et al., 2005; Repping et al., 2006; Karafet et al., 2008). The Y consortium published the Y chromosome phylogenetic tree topology based on 243 SNP markers (Consortium, 2002). A further 351 markers were used to develop a better-resolved tree (Karafet et al., 2008).

In the current era of rapid data generation traditional and more recently Massive Parallel Sequencing (MPS) platforms have been used, which together with the development of extremely powerful software have started to provide us with new markers and lineages. Hallast et al. (Hallast and Jobling, 2017) reported 13,621 SNPs after resequencing 3.7 Mb of the Male Specific Region of Y Chromosome (MSY). However, many thousands have been detected by others as well using Massive Parallel Sequencing techniques, increasing the potential of the Y chromosome as an evolutionary and human identification tool (Francalacci et al., 2013; Poznik et al., 2013; Wei et al., 2013; Scozzari et al., 2014). These developments are helping to determine new directions in human population migrations and evolution.

This Research Topic on the “Role of the Y Chromosome in Molecular Anthropology, Forensics, and Genetic Genealogy” aims to generate a review of the role of various Y markers, bringing to light current developments and applications within the field.

Male and female humans have contributed unequally to geographic expansion due to biological and behavioural differences. Dispersive genetic forces act quickly on uniparental sequences, which leads to changes in sequences, with one population branching off from the other. Mutations at the AZFc region or DYS448, which are regional specific or to some extent ethnic group-specific, are examples (Adnan et al., 2018; Adnan et al., 2021). These haplogroups were previously predicted using SNPs, which was a conventional method, but now these haplogroups can be successfully predicted from Y-STRs using different software packages like Nevgen and Whit Athey’s Haplogroup Predictor Tool (Athey, 2006). These software packages render quite precise information, which is supported by SNPs typing results. This might be due to STRs being more polymorphic as compared to SNPs (He et al., 2019). Studies on Y-STRs are still ongoing with several new applications. Ravasini et al. have identified four phylogenetically related samples with a null allele at DYS448 and a tetrallelic pattern at DYF387S1, two Y-STRs located in the AZFc. Through MPS analysis, they found that the unusual Y-STR pattern may be due to a 1.6 Mb deletion arising concurrently or after a 3.5 Mb duplication event. Jordamovic et al., predicted the Y haplogroup using Whit Athey’s Haplogroup Predictor, based on haplotype data on Powerplex Y 23 -STR markers. They found that Athey’s Haplogroup Predictor offers more accurate results and a higher probability of detecting rare haplogroups as compared to SNPs results. Luo et al. have characterized the genetic structure and forensic parameters of the She and Hakka ethnic groups from Guangdong province of China. They ascertained that Guangdong Hakka has a close relationship with Southern Han, and the genetic pool of Guangdong Hakka was influenced by closely located Han populations. The predominant haplogroups of the Guangdong She group were O2-M122 and O2a2a1a2-M7, while Guangdong She clustered with other Tibeto-Burman language-speaking populations.

Bini et al. studied the relationships between old (16–18th century remains) and modern male populations of a population isolate from Roccapelago, which is a small village located in the Northern Central Apennines in Italy. In total, 14 modern samples and 25 ancient mummies were genotyped. They utilized several techniques to ascertain relationships between the old and modern-day populations from this area. Y haplogroup predictor tool (Athey, 2006) and Nevgen software (Cetkovic Gentula, 2015) were used to infer Y haplogroups from Y-STR haplotypes, which showed a close relationship between old and modern samples. Network analysis showed relationships between the two sets of samples.

The combined employment of various forensic genetics and archaeogenetic techniques demonstrates the value of a multidisciplinary approach, which is perhaps the way forwards for human Y chromosome analysis. Indeed, the Y chromosome continues to march on.
